# Crop insurance as a climate risk management tool: Evidence from three districts of Punjab, Pakistan

**DOI:** 10.1371/journal.pone.0344460

**Published:** 2026-03-18

**Authors:** Muhammad Khan, Kabir Ahmad Sidhu, Yasir Majeed Waraich, Wafa Ghardallou

**Affiliations:** 1 Centre of Excellence in Competition Law, Competition Commission of Pakistan, Islamabad, Pakistan; 2 Securities and Exchange Commission of Pakistan (SECP) NIC Building, Islamabad, Pakistan; 3 Department of Social Sciences, IQRA University, Islamabad, Pakistan; 4 Department of Accounting, College of Business Administration, Princess Nourah bint Abdulrahman University, Riyadh, Saudi Arabia; Universitas Airlangga, INDONESIA

## Abstract

Pakistan’s agricultural sector is increasingly vulnerable to climate change–induced shocks, posing serious risks to farm incomes and rural livelihoods. Although crop insurance has been promoted as a key risk-mitigation instrument, and several public and private schemes have been introduced—particularly in Punjab—farmer participation remains strikingly low. This study provides one of the first systematic assessments of farm households’ perceptions of climate-related risks, their awareness of crop insurance, and the coping mechanisms they adopt in response to climatic shocks. The analysis draws on primary survey data from 324 farmers across 27 mouzas (villages) in the districts of Bahawalpur, Gujrat, and Faisalabad. Descriptive findings reveal a pronounced perception–adoption gap: while 84% of respondents view climate hazards as a major threat to agriculture, only 21% are aware of crop insurance schemes, and fewer than 3% have ever purchased coverage. In the absence of formal insurance, farmers predominantly rely on informal credit networks, valued for their accessibility, flexibility, and trust-based nature. Econometric estimates using logit and probit models indicate that education and financial inclusion significantly increase the likelihood of insurance awareness, underscoring the central role of financial literacy in shaping adaptation decisions. Overall, the results highlight a substantial disconnect between farming communities and formal risk-management institutions in Pakistan. The study emphasizes the need for localized awareness campaigns, simplified enrolment procedures, credible institutional delivery mechanisms, and innovative public–private partnerships to position crop insurance as an effective resilience-building tool in Pakistan’s climate-vulnerable agricultural sector.

## 1. Background and introduction

Pakistan is recognized as one of the countries most vulnerable to the impacts of climate change due to its geographic location, climatic variability, and socio-economic fragility [1]. Despite contributing minimally to global greenhouse gas emissions, the country consistently ranks among the top five nations most affected by climate change [2]. This heightened vulnerability has manifested through recurring natural disasters—ranging from floods, droughts, and heatwaves to landslides—while the nation’s adaptive capacity remains critically limited [3]. Over the past three decades, Pakistan has faced a series of climate-induced catastrophes, including the prolonged drought of 1999–2003, the historic floods between 2010 and 2014, the devastating floods of 2022, and the ongoing inundations in 2025 [4]. Projections indicate that by 2050, the country’s average temperature could rise by 2–3°C, accompanied by significant variations in rainfall distribution [5].

The economic impacts of climate change on Pakistan are severe. In the absence of effective adaptation, annual losses could reach up to 6% of GDP by 2050, mainly through damage to agriculture, water resources, infrastructure, and public health [1,6]. The magnitude of climate-related risks is amplified by Pakistan’s agrarian economy, where agriculture contributes about 22% of GDP and employs over 37% of the labor force [7]. It is also the country’s largest export sector, generating nearly 75% of total export earnings [8], while simultaneously meeting the food needs of a rapidly growing population [9]. In addition to ensuring food supply, agriculture underpins industrial activity by supplying essential raw materials to key sectors, including textiles, sugar, and vegetable oil production [10].

Climate change is exerting significant pressure on agricultural productivity in Pakistan, a sector heavily dependent on irrigation [11]. Shifts in temperature and precipitation patterns are altering crop growth cycles, reducing yields, and affecting overall quality, while extreme events such as floods and droughts frequently damage both crops and rural infrastructure [12]. The World Bank [13] warns that Pakistan’s vulnerability to such climate-induced hazards threatens not only agricultural sustainability but also food security and rural livelihoods. The implications of climate-related disasters are particularly pronounced in Punjab, where agriculture—including crops, livestock, fisheries, and forestry—accounts for 26% of provincial GDP and provides employment to 40% of the population. The World Bank [7] reports that between 1973 and 2018, natural disasters—predominantly floods—affected an average of three million people annually in Punjab alone. As Pakistan’s most densely populated province, Punjab comprises 36 administrative districts and spans 205,344 square kilometers. A significant share of this land is arable, making it one of the world’s most intensively cultivated and irrigated regions. Out of Punjab’s 16.5 million hectares of cultivated land, 14.3 million hectares—about 87%—are irrigated, underscoring its central role in Pakistan’s agricultural production. Several major tributaries of the Indus River flow through the province from north to south, supporting extensive irrigation networks. Punjab is also Pakistan’s breadbasket, producing 20 million tons of wheat—76% of national output—in 2021–22 [14]. Consequently, climate-induced shocks to rural livelihoods in Punjab can trigger wider economic and political instability and deepen poverty across Pakistan.

Against this backdrop, a critical question is how to protect vulnerable communities in Pakistan from the adverse effects of climate change. To answer this question, previous literature on climate change and agricultural productivity has focused on mitigation strategies [15], adaptation measures [9], and impact assessments [16]. Moreover, [17] note that climate change presents a systemic risk to the local communities, destroying social networks and creating widespread human suffering through loss of livelihoods and displacement [18]. Dealing with these bio-physical shocks require insurance coverage by public and private insurance companies.

The empirical literature also supports a positive relationship between crop insurance and farmers income [19,20]. However, farmers in developing countries are not inclined towards insurance policies in general. For instance, Sun et al., [21] conducted an empirical analysis in China to explore the relationship between climate change and farmers’ willingness to purchase agricultural insurance. Their empirical findings reveal no significant relationship between the two variables. This is a surprising outcome given existing research has long underscored the critical role of crop insurance in ensuring agricultural resilience and safeguarding production in the face of climate-related challenges [22].

This study aims to address this puzzle by examining the farmers’ awareness of climate-related threats and their perception of the potential role of crop insurance to deal with these challenges. To the best of our knowledge, it is the first attempt to gauge the crop insurance awareness of farming community in this vulnerable part of the world and understand their viewpoint on using it as an adaptation tool for climate-related risks. In addition, our study also seeks to understand the reasons behind limited participation of farmers in the existing insurance schemes. By analyzing both perceptions and barriers, the study seeks to provide insights into how crop insurance could be made a more effective tool for building resilience against climate risks in Pakistan’s agrarian economy. For our primary data analysis, we selected the sample from Punjab, taking Gujrat, Faisalabad and Bahawalpur as our representative districts. Our main outcomes show very limited awareness among the farming community regarding the availability of crop insurance. The results also reveal an important role of education in using crop insurance to combat climate change and improving adaptive decision making.

The remainder of the study is structured as follows: Section 2 presents crop insurance in Pakistan at national and provincial levels. Section 3 presents our data and the adopted methodology. Section 4 reports our main outcomes and, finally, section 5 offers conclusions and policy recommendations.

## 2. Crop insurance efforts in Pakistan

Crop insurance is an important tool for farmers around the world to manage agriculture risks, such as natural disasters, disease outbreaks, and fluctuations in commodity prices. As reported by Food and Agriculture Organization [23] of the United Nations, crop insurance can play a crucial role in these situations by reducing the impacts of natural disasters on agriculture. The study examines Asian countries and recommends that governments in the region should invest in strengthening their agricultural insurance systems including increasing public funding, improving risk management practices, and developing more effective information systems. Taking the case of Indian economy, Ranganathan et al., [24] note that crop insurance can help reduce poverty and improve food security in South Asian countries, as farmers are able to manage the financial risks associated with agriculture and invest in more productive technologies and inputs. While acknowledging the importance of crop insurance, Government of Pakistan has introduced the following schemes at both national and provincial levels:

a) **Crop Loan Insurance Scheme:** This scheme is offered by the State Bank of Pakistan and provides insurance coverage for crop loans taken out by farmers. The insurance covers the outstanding loan balance in the event of crop damage or loss due to natural calamities or other events [25]
b) **Takaful Crop Insurance:** This is an Islamic crop insurance scheme offered by Pakistan Takaful Company Limited. The insurance is based on the concept of mutual assistance and provides coverage for crop damage or loss due to natural calamities or other events.
c) **Weather-Based Crop Insurance**: This is a relatively new type of crop insurance that uses weather data to determine insurance payouts. The insurance covers losses due to weather-related events such as drought, excessive rainfall, or temperature extremes [26].
d) **Livestock Insurance Scheme:** In addition to crop insurance, there is also a livestock insurance scheme available in Pakistan. This scheme provides coverage for losses due to the death of livestock, including cattle, buffalo, and goats [27].

As evident from the above description, the availability of crop insurance is not the primary challenge, as a variety of schemes exist to protect farmers against climate shocks that can severely affect crops and livestock. However, the effectiveness and acceptability of these schemes vary considerably and are influenced by factors such as premium levels, coverage terms, and the ease of claim processing. Against this background, the present study hypothesizes and tests the role of farmers’ awareness in their decision to get insurance scheme. We argue that the success and uptake of any insurance scheme is largely determined by farmers’ awareness. Without adequate awareness, farmers are unable to assess, compare, and make informed decisions about the costs and benefits of available insurance options. This issue is especially pronounced in countries like Pakistan, where a large proportion of the farming population is illiterate and/or lacks basic financial literacy, limiting their ability to evaluate the financial viability and suitability of available crop insurance schemes [28].

### 2.1. Crop insurance schemes in Punjab

As noted earlier, given the province’s heavy reliance on agriculture, farmers remain highly exposed to climate-induced risks such as floods, droughts, and pest outbreaks. To address these vulnerabilities, the Government of Punjab has introduced a series of crop insurance programs aimed at safeguarding farm incomes and providing financial protection in the event of crop loss or damage. The following discussion outlines the key schemes launched in the province and evaluates their role in managing agricultural risks.

A. **Prime Minister’s Agriculture Emergency Program (PMAEP)** – Crop Insurance Scheme was launched in 2018 under the federal government’s Agriculture Emergency Program. This scheme was designed to protect farmers against losses caused by natural disasters, including floods, droughts, hailstorms, and crop diseases [29]. The insurance aimed to reduce financial vulnerability for smallholders, encouraging investment in better seeds and fertilizers. The key feature of this crop insurance scheme was the provision of 50–70% premium subsidies for small farmers, making insurance affordable. Compensation was provided if crop yields fell below a predetermined threshold. Overall, 500,000 farmers in Punjab were enrolled by 2022. However, many farmers remain unaware of the scheme’s benefits, and bureaucratic hurdles have slowed compensation disbursement.
B. **Punjab Crop Insurance Scheme (PCIB):** This is a government-sponsored crop insurance scheme that provides coverage for a variety of crops and covers losses due to natural calamities, pests and diseases, and other events [30]. The Punjab government, in collaboration with private insurers, introduced PCIP in 2020 to enhance risk coverage. The payouts were triggered if the average yield in a designated area (e.g., a tehsil or district) fell below a set benchmark. The scheme used geographic information systems and remote sensing to assess crop damage, reducing fraud. The scheme was based on a private partnership and engaged EFU General Insurance and TPL Insurance to administer the scheme. By 2023, the scheme expanded coverage to over 1 million acres and aimed to improve trust among farmers because of a transparent damage assessment mechanism. The scheme had limited coverage as only major crops were included, leaving out vegetables and fruits, and due to high operational costs, it faced difficulties in verifying small-scale claims [31]
C. **Weather-Based Crop Insurance:** This is a relatively new type of crop insurance that uses weather data to determine insurance payouts. The insurance covers losses due to weather-related events such as drought, excessive rainfall, or temperature extremes. Supported by the World Bank and the Punjab government, this scheme aimed to address shortcomings of traditional crop insurance [32]. The automated payouts used weather stations and satellite data to trigger payouts based on rainfall, temperature, and humidity levels. It was planned that through quick disbursements, the farmers would receive compensation within weeks of a weather event. Although the scheme benefitted over 200,000 farmers by 2023, it faced challenges since limited weather stations led to inaccuracies, and farmers’ skepticism led to distrust of automated systems.
D. **Punjab Kissan Insurance Scheme:** This scheme provides insurance coverage for farmers who have taken out loans from the Punjab Provincial Cooperative Bank. The insurance covers the outstanding loan balance in the event of crop damage or loss due to natural calamities or other events. Table 1 summarizes the main crop insurance schemes in Punjab along with their coverage, payments mechanism and key challenges.

**Table 1:** Comparative analysis of crop insurance schemes in Punjab.

Scheme	Launch Year	Coverage	Payout Mechanism	Key Challenges
Prime Minister’s Agriculture Emergency Program (PMAEP)	2018	Wheat, Rice, Cotton	Yield-based assessment	Low awareness, delays
Punjab Crop Insurance (PCIP)	2020	Major crops	Area-yield index	Limited crop variety
Weather Index (WIBCI)	2020	Drought-prone areas	Automated weather triggers	Data reliability issues
Livestock & Crops (LCIS)	2021	Fodder crops	Biometric verification	Low farmer participation

In conclusion, following the footprints of the Federal Government, the Government of Punjab has also introduced several crop insurance programs to help farmers manage the risks associated with agriculture, including the Punjab Agricultural Insurance Company Limited (PAIC), Weather-based Crop Insurance, and the Agriculture Emergency Programs. These programs offer insurance coverage for a range of crops and provide compensation in case of crop losses due to natural disasters and other risks. However, there is limited research on farmers’ views, perceptions, and practices regarding crop insurance schemes. Therefore, this research aims to bridge this data gap by exploring the rural farm households’ awareness and perception about crop insurance in general and their current practices in particular.

## 3. Conceptual framework

While the importance of crop insurance as a hedging tool is widely acknowledged in the literature, a key challenge in developing countries such as Pakistan lies in improving farmers’ perceptions of insurance and, consequently, their participation in such schemes. To address this issue, the conceptual framework of this study draws on climate risk perception, financial inclusion, education, and innovation-adoption theory [12]. We argue that greater awareness of climate change enhances farmers’ readiness to undertake adaptive actions. A better understanding of climate-related risks is therefore expected to generate demand for formal risk-mitigation instruments, including crop insurance, while education enables farmers to assess and compare the benefits of different insurance schemes. The level of education thus conditions farmers’ participation in insurance initiatives implemented by federal and provincial governments.

Likewise, financial inclusion constitutes a critical channel for benefiting from these insurance schemes, as most crop insurance programs in Pakistan are delivered through formal banking systems. Access to formal credit is also essential for obtaining affordable loans to meet seasonal investment needs. Beyond these individual-specific factors, common constraints—such as bureaucratic hurdles in accessing insurance products as well as cultural and religious considerations—may further influence farmers’ cost–benefit evaluations of available insurance options. Together, these factors shape insurance awareness and ultimately determine farmers’ participation in existing crop insurance schemes.

Addressing the gap between the availability of insurance schemes and their low uptake among farmers can enhance the outreach of these programs and integrate excluded farming communities into formal insurance safety nets. These theoretical considerations guide the formulation of the study objectives and the design of the survey instrument, ensuring that the empirical analysis is grounded in a coherent, well-structured analytical framework.

## 4. Materials and methods

The research employed a mixed methods approach to achieve complementarity and minimize the limitations of any single method. The primary data collection included semi-structured interviews with rural farm households in three districts to collect quantitative and qualitative information. Wherever possible, secondary data from existing literature were used to triangulate and substantiate the primary data.

### 4.1. Target population

As identified in the background section, small-scale farmers are more reluctant to opt for crop insurance. Therefore, the target population of the survey includes rural households in the three districts of Punjab that are engaged in agriculture as farmers. Landlords or corporate farmers were not included in the target population.

### 4.2. Sampling

A two-stage stratified cluster random sampling design was used for the rural farm household survey of 324 households from 27 Mouzas (villages) in three districts of Punjab. In the first stage, a pre-determined number of mouzas (nine) from each district were selected. The list of mouzas prepared in the 2017 population census was used as a sampling frame for the selection of sample mouzas at the first stage.

At the second stage, from each sampled Mouza, 12 farm households were selected using the random walk method and right-hand rule. A sample size of 324 rural farm households from 27 mouzas (i.e., 12 household surveys in one Mouza) from three districts was completed. The social science research formula [33] suggests interviewing 273 respondents to obtain results that have a confidence level of 90% and a 5% margin of error. This sampling estimate was increased by 20% to incorporate non-response and interviewing errors; therefore, a total of 324 interviews were undertaken in three sampled districts.

### 4.3. Data collection

Face-to-face interviews were conducted with farmers in the targeted sample households. Each interview included a brief introduction about the purpose and scope of the interview. The information obtained was verbal and reported by the interviewer on the questionnaire.

The research followed ethical standards, including obtaining informed consent, explaining each question in the local language according to respondents’ comprehension, and ensuring respondent privacy.

**Fig 1:**
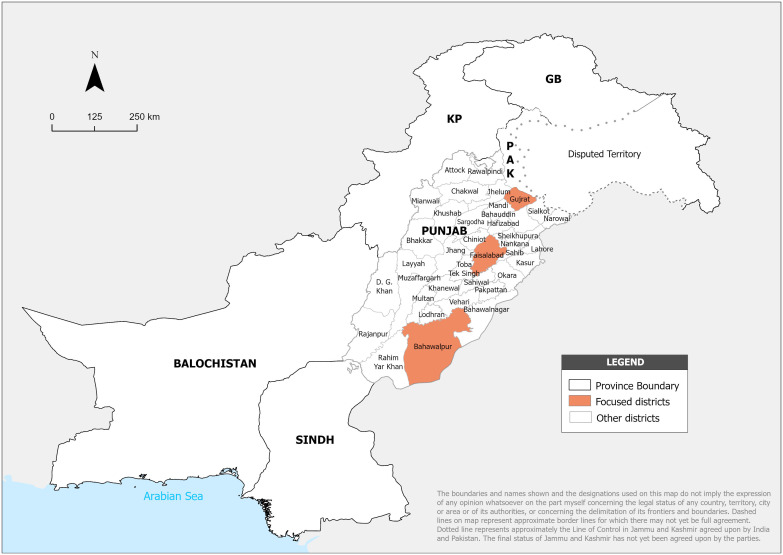
Study area map.

In total, 324 interviews were conducted in the Gujrat, Faisalabad, and Bahawalpur districts. These districts were selected because of a) coverage of the southern, central, and northern sides of Punjab and b) ease of data collection, as the researcher used his local contacts in these areas to complete the surveys. Before deployment, all seven people involved in data collection were thoroughly trained on research purposes, ethical considerations, interviewing techniques, and data collection tools. Table 2 presents the distribution of interviews across the sampled districts. As shown, the number of interviews is identical in all three districts, ensuring equal representation across the selected study areas (Fig 1).

**Table 2:** Number of interviews by district.

District	Number of sampled Mouzas	Number of interviews in each Mouza	Total Interviews
Bahawalpur	9	12	108
Gujarat	9	12	108
Faisalabad	9	12	108
Total Interviews	27	–	324

### 4.4. Survey instrument

We used a structured questionnaire administered through face-to-face interviews by trained enumerators who spoke the local language. Before beginning each interview, informed consent was obtained, and enumerators read every question aloud to ensure clarity and accuracy. Our sampling followed a two-stage stratified cluster design: nine mouzas (villages) were selected from each of the three districts, Bahawalpur, Faisalabad, and Gujrat, and twelve farm households were interviewed in each mouza, resulting in a total sample of 324 respondents.

The data were collected with prior consent from the participants. The questionnaire gathered information on household demographics, land and livestock ownership, perceived production risks, familiarity with and experience of insurance, borrowing behavior, and interactions with banking services. We piloted the instrument in a non-sample mouza to refine question wording and improve skip patterns. Enumerators received training on research ethics, effective probing, and the use of standardized explanations for key concepts such as crop insurance, ensuring consistency across all interviews.

### 4.5. Data analysis

Our empirical analysis is guided by the overall research problem and covers all aspects of the research questions. In the first step, we check the accuracy and completeness of rural farm households’ survey data and code survey responses against the desired outcomes and other variables.

In the second step, we perform initial analysis using descriptive statistics, including cross-tabulations and frequencies, to identify patterns and directional trends in the data.

In the subsequent step, we perform regression analysis to determine the strength of any association between crop insurance awareness and relevant independent variables while controlling for other variables. For this purpose, we rely on logistic regression (logit) which is usually used when the dependent variable is dichotomous [9]. Logistic regression measures the relationship between the dichotomous dependent variable and one or more independent variables by estimating probabilities using a logistic function. Logistic regression is helpful when the objective is to predict the presence or absence of a characteristic or outcome based on values of a set of independent (or predictor) variables.

For robustness purposes, we also use a probit model to check whether our main variables are robust to model specification.

Our theoretical specification highlighting the role of socio-economic features in insurance awareness takes the following form:


AWR=f(EDU,LH, PREM,BANK, D.BAH,D.GUJ,D.FAIS) (1)

Here, the dependent variable, AWR indicates whether the respondent knew about the crop insurance scheme (1 = Yes, 0 = No). DUM.BAH and DUM.GUJ show dummy variables for Bahawalpur and Gujrat with Faisalabad serving as baseline district. EDU is human capital dummy taking the value 1 for primary and above, 0 otherwise. LH shows land holding size is acres, PREM is maximum premium (in PKR) per season per acre household is willing to pay, BANK represents whether the respondent used any banking services (1 if yes and 0 if not). The econometric model based on the above equation takes the following form:


$$AWR=\propto_{\text{0}}+\propto_{\text{1}}DUM.BAH+\propto_{\text{2}}DUMGUJ+\propto_{\text{3}}EDU+\propto_{\text{4}}LH+\propto_{\text{5}}PREM+\propto_{\text{6}}BANK+\varepsilon$$ (2)

While the study relies on many variables to capture individuals’ socioeconomic conditions, the regression models are based on the principle of parsimony and utilize only the most relevant variables covering respondents’ education, land holding, willingness to pay premium, financial inclusion and geographical differences.

a) **Awareness measure (operational definition):**
  - Respondents were asked: “Do you know about the crop insurance scheme?” (Yes/No). We code Awareness_i = 1 if “Yes,” 0 otherwise.
  - This single-item indicator provides a conservative interpretable measure of awareness aligned with the study objective of benchmarking baseline knowledge. Related items (current insurance holdings and trusted providers) are used descriptively to contextualize awareness but are not included in a composite score.

Models are then cross-checked with frequencies to ensure that regression results reflect overall trends and are not one-off, circumstantial instances of correlation. Where regression analysis is not appropriate (for example, in analyzing outcomes against each other), the study uses a two-by-two crosstabulation with correlation tests to determine the strength of association between outcomes.

## 5. Results and discussion

The research findings are presented in a structured sequence. First, respondents’ profiles are described, followed by an analysis of their perceptions of threats to crops to establish the contextual background. Next, the study examines respondents’ awareness of crop insurance schemes, borrowing practices, and experiences with the banking sector. Finally, the results related to the research hypotheses are reported to identify factors influencing access to and uptake of banking services and crop insurance.

All variables are analysed at the district level and, where relevant, by education level. The results tables report both counts and percentages because, in some cases, the number of observations in specific cells is small, and percentages alone could be misleading. The paper addresses four research questions sequentially. The first research question examines perceived climate risks (Table 3). The second assesses awareness and willingness to pay (Tables 4–6). The third explores correlates of awareness (Table 7). The final research question identifies barriers to engagement, synthesized from borrowing patterns and exposure to the banking sector.

**Table 3:** Respondents’ views about the significant threat to crops by district.

	Bahawalpur		Faisalabad		Gujrat		Overall	
**N**	**%age**	**N**	**%age**	**N**	**%age**	**N**	**%age**
**Weather extremes (temperature, heavy rains, floods, hail, heat waves, windstorms)**	53	49.1%	40	37.0%	78	72.0%	171	52.8%
**Drought**	34	31.5%	14	13.0%	7	6.5%	55	17.0%
**Pests and diseases**	81	75.0%	99	91.7%	91	84.3%	271	83.6%
**Decline in crop prices**	5	4.6%	6	5.6%	3	2.8%	14	4.3%
**Wildfires**	1	0.9%	0	0.0%	1	0.9%	2	0.6%
**Salinity**	5	4.6%	0	0.0%	0	0.0%	5	1.5%
**Low-quality pesticides**	11	10.2%	10	9.3%	7	6.5%	28	8.6%
**Weed**	3	2.8%	3	2.8%	2	1.9%	8	2.5%
**Other (specify)**	8	7.4%	17	15.7%	5	4.6%	30	9.3%

**Table 4:** Respondents’ knowledge and experience with insurance schemes by district.

		Bahawalpur		Faisalabad		Gujrat		Overall	
		N	%age	N	%age	N	%age	N	%age
**Do you know about the crop insurance scheme?**	Yes	31	28.7%	12	11.1%	24	22.2%	67	20.7%
	No	77	71.3%	96	88.9%	84	77.8%	257	79.3%
**Do you have any insurance now?**	Yes	16	14.8%	6	5.6%	15	13.9%	37	11.4%
	No	92	85.2%	102	94.4%	93	86.1%	287	88.6%
**What type of insurance do you have?**	Life	13	81.3%	6	100.0%	14	93.3%	33	89.2%
	Health	3	18.8%	0	0.0%	0	0.0%	3	8.1%
	Livestock	0	0.0%	0	0.0%	0	0.0%	0	0.0%
	Crop	1	6.3%	0	0.0%	0	0.0%	1	2.7%
	Other	0	0.0%	0	0.0%	1	6.7%	1	2.7%
**Who do you trust to buy you insurance? Other**	Banks	3		3		1		7	
	Pakistan post	0		0		2		2	
	Private Insurance company	11		2		8		21	
	Government Insurance Company	1		1		4		6	
	Other	0		0		0		0	

**Table 5:** Respondents’ views about the government’s role in crop insurance schemes by district.

		Bahawalpur		Faisalabad		Gujrat		Overall	
		N	%age	N	%age	N	%age	N	%age
**Do you feel Govt. should provide crop insurance against weather events or natural disasters?**	Yes	90	83.3%	84	77.8%	95	88.0%	269	83.0%
	No	18	16.7%	24	22.2%	13	12.0%	55	17.0%
**If yes, what type of crop do you want to be insured?**	Total	108	100.0%	108	100.0%	108	100.0%	324	100.0%
	Cereals/Grains	73	67.6%	83	76.9%	92	85.2%	248	76.5%
	Fiber crops	76	70.4%	5	4.6%	1	0.9%	82	25.3%
	Pulses	0	0.0%	0	0.0%	1	0.9%	1	0.3%
	Vegetables	0	0.0%	8	7.4%	1	0.9%	9	2.8%
	Fruits	4	3.7%	1	0.9%	9	8.3%	14	4.3%
	Oilseeds	15	13.9%	2	1.9%	3	2.8%	20	6.2%
	Other	1	0.9%	2	1.9%	3	2.8%	6	1.9%
**How much maximum premium per season per acre will you pay (PKR) for the insurance?**		1,241 PKR		1,083 PKR		1,839 PKR		1,403 PKR	

**Table 6:** Respondents’ views about the government’s role in crop insurance schemes by level of education.

		Illiterate		Up to Matriculation		Intermediate & above		Overall	
		N	%age	N	%age	N	%age	N	%age
**Do you feel Govt. should provide crop insurance against weather events or natural disasters?**	Yes	107	84.9%	124	82.1%	38	80.9%	269	83.0%
	No	19	15.1%	27	17.9%	9	19.1%	55	17.0%
**If yes, what type of crop do you want to be insured?**	Cereals/Grains	97	77.0%	115	76.2%	36	76.6%	248	76.5%
	Fiber crops	43	34.1%	34	22.5%	5	10.6%	82	25.3%
	Pulses	0	0.0%	1	0.7%	0	0.0%	1	0.3%
	Vegetables	2	1.6%	7	4.6%	0	0.0%	9	2.8%
	Fruits	5	4.0%	7	4.6%	2	4.3%	14	4.3%
	Oilseeds	13	10.3%	7	4.6%	0	0.0%	20	6.2%
	Other	1	0.8%	5	3.3%	0	0.0%	6	1.9%
**How much maximum premium per season per acre will you pay (PKR) for the insurance?**		973 PKR		1452 PKR		2330 PKR		1403 PKR	

**Table 7:** Matrix of correlations.

Variables	(1)	(2)	(3)	(4)	(5)	(6)	(7)	(8)
(1) AWR	1.000							
(2) EDU	0.086	1.000						
(3) LH	0.097	0.122	1.000					
(4) PREM	0.164	0.121	−0.020	1.000				
(5) LH	0.193	0.197	0.079	0.135	1.000			
(6) D.BAH	0.140	−0.207	0.047	−0.062	−0.164	1.000		
(7) D.GUJ	0.027	0.089	0.088	0.131	0.082	−0.500	1.000	
(8) D.FAIS	−0.167	0.118	−0.135	−0.069	0.082	−0.500	−0.500	1.000

### 5.1. Socio-demographic profile of respondents

The socio-demographic profile of respondents provides a broader snapshot of their characteristics and enables readers to better understand and interpret results. These characteristics are summed up in Table 8. As can be viewed, a total of 324 rural farmers were interviewed during primary data collection, with an equal split across the Bahawalpur, Faisalabad, and Gujrat districts. The average age of respondents was 46 years, and nearly 80% of them were the head of households or sons/daughters of household heads. The reason for interviewing the head of the household or lead farmer was to understand crop-related financial aspects in detail. The average household size was nine people.

**Table 8:** Respondent’s basic characteristics.

		Bahawalpur		Faisalabad		Gujrat		Overall	
		N	%age	N	%age	N	%age	N	%age
		108	100%	108	100%	108	100%	324	100%
**Respondent age (in completed years)**		46 years		45 years		47 years		46 years	
**Respondent’s relation to the head of household**	Head	91	84.3%	84	77.8%	75	69.4%	250	77.2%
	Son/Daughter	13	12.0%	19	17.6%	24	22.2%	56	17.3%
	Brother/sister	4	3.7%	3	2.8%	3	2.8%	10	3.1%
	Father/mother	0	0.0%	2	1.9%	6	5.6%	8	2.5%
**Educational level of the respondent?**	Completely illiterate	38	35.2%	18	16.7%	12	11.1%	68	21.0%
	No formal education, yet can read	2	1.9%	1	0.9%	6	5.6%	9	2.8%
	Below primary	3	2.8%	2	1.9%	5	4.6%	10	3.1%
	Primary	21	19.4%	18	16.7%	10	9.3%	49	15.1%
	Middle	19	17.6%	23	21.3%	28	25.9%	70	21.6%
	Matriculation	19	17.6%	26	24.1%	26	24.1%	71	21.9%
	Intermediate	3	2.8%	12	11.1%	6	5.6%	21	6.5%
	Graduation	1	0.9%	3	2.8%	1	0.9%	5	1.5%
	Post-graduation	2	1.9%	5	4.6%	14	13.0%	21	6.5%
**How many people usually live and eat in the household? (Do not list guests or visitors)**		8 people		8 people		9 people		9 people	

One in five respondents was illiterate, this proportion was highest in Bahawalpur and lowest in Gujrat. More than 70% of respondents had primary or above education level. Farmers from Gujrat were more educated than those from other districts, particularly those in Bahawalpur.

The research identified and interviewed rural households that had farming experience. The respondents were probed using multiple-response questions to enlist a range of landholding options. The outcomes of this inquiry are reported in Table 9. As can be viewed, a large majority (85%) of respondents had their own land, with Gujrat respondents owning more land, followed by Faisalabad and Bahawalpur. A little over 44% of respondents had rented land, and this practice appeared more visible in Faisalabad than in other districts. More respondents from Gujrat (10.2%) reported renting out their land compared to the average of 8.3%. On average, each rural farm household owned 15.14 acres of land, with Faisalabad respondents owning the fewest acres of land (10.99 acres). Almost all the respondents owned livestock, mostly cows and buffalo, while about one-third also owned goats.

**Table 9:** Households’ land and livestock holdings.

		Bahawalpur		Faisalabad		Gujrat		Overall	
		N	%age	N	%age	N	%age	N	%age
**Agricultural land holding size of household (read response)?**	Owned	84	77.8%	95	88.0%	98	90.7%	277	85.5%
	Culturable Waste	2	1.9%	0	0.0%	6	5.6%	8	2.5%
	Rented in	49	45.4%	55	50.9%	39	36.1%	143	44.1%
	Rented out	8	7.4%	8	7.4%	11	10.2%	27	8.3%
	Shared in	6	5.6%	9	8.3%	2	1.9%	17	5.2%
	Shared out	0	0.0%	3	2.8%	1	0.9%	4	1.2%
	Other	0	0.0%	1	0.9%	4	3.7%	5	1.5%
**Household landholding size**		16.58 acres		10.99 acres		17.84 acres		15.14 acres	
**Does the household own livestock?**	Yes	98	90.7%	104	96.3%	101	93.5%	303	93.5%
	No	10	9.3%	4	3.7%	7	6.5%	21	6.5%
**Select the primary livestock your household raises.**	Cows	87	80.6%	80	74.1%	67	62.0%	234	72.2%
	Buffalos	55	50.9%	94	87.0%	90	83.3%	239	73.8%
	Goats	47	43.5%	35	32.4%	25	23.1%	107	33.0%
	Sheep’s	2	1.9%	4	3.7%	5	4.6%	11	3.4%
	Other	0	0.0%	2	1.9%	6	5.6%	8	2.5%

### 5.2. Respondents’ perception of threats to crops

An important aspect of this study is to examine rural farm households’ understanding of potential threats to crops. The first multiple-response question asked respondents to list significant threats to their crops. Pests and disease attacks appeared as a key concern across all districts, as 83.6% of respondents mentioned this as a threat. Respondents from Faisalabad perceived this issue as more severe (91.7%) compared to Bahawalpur, where only 75% identified it as a significant threat.

The reality of climate change is clearly reflected in the study’s findings, with 52.8% of respondents identifying rising temperatures, heavy rainfall, floods, hailstorms, heatwaves, and windstorms as major threats. Bahawalpur District, located in Southern Punjab, comprises desert regions as well as areas with limited access to water. Consequently, a substantially higher proportion of respondents from Bahawalpur (31.5%) perceived drought as a significant threat—almost twice the proportion reported by households in Faisalabad and Gujrat districts. Overall, a large majority believe pests, diseases, and extreme weather conditions as significant threats to their crops. These results are in line with the previous literature that reports farmers awareness of climate related risks in Pakistan [34]. This finding is important given the fact that farmers’ wrong beliefs could lead to wrong adaptation strategies exacerbating the effects of climate change [35].

### 5.3. Respondent’s awareness of crop insurance

After exploring respondents’ perceptions of crop-related threats, the next set of questions examined their knowledge and experience with crop insurance schemes. Overall, just over 20% of respondents were aware of such schemes, with awareness highest in Bahawalpur, followed by Gujrat. In Faisalabad, only 11% reported having any form of insurance at the time of the interview. Life insurance emerged as the most common type across districts, while health insurance—reported exclusively in Bahawalpur—was less prevalent. Interestingly, most respondents expressed greater trust in private insurance providers, which aligns with the fact that life insurance is predominantly offered by the private sector. Overall, the findings indicate that the farming community has limited awareness of crop insurance schemes available at both federal and provincial levels. This lack of knowledge represents a significant barrier to accessing formal risk protection, leaving farmers vulnerable to biophysical shocks that informal arrangements and social networks cannot adequately mitigate [17].

While analyzing the data by respondents’ level of education, we noticed that respondents with intermediate and above education had 15 percentage points more knowledge about crop insurance compared to others. Table 10 reports the association between respondents’ education and knowledge of insurance schemes. Although the number of observations was smaller, another interesting aspect is that respondents with intermediate and above educational levels preferred government insurance companies compared to those with relatively less education.

**Table 10:** Respondents’ knowledge and experience with insurance schemes by level of education.

		Illiterate		Up to Matriculation		Intermediate & above		Total	
		N	%age	N	%age	N	%age	N	%age
**Do you know about the crop insurance scheme?**	No	108	85.7%	119	78.8%	30	63.8%	257	79.3%
	Yes	18	14.3%	32	21.2%	17	36.2%	67	20.7%
**Do you have any insurance now?**	No	117	92.9%	131	86.8%	39	83.0%	287	88.6%
	Yes	9	7.1%	20	13.2%	8	17.0%	37	11.4%
**What type of insurance do you have?**	Life	7	77.8%	18	90.0%	8	100.0%	33	89.2%
	Health	2	22.2%	1	5.0%	0	0.0%	3	8.1%
	Crop	0	0.0%	1	5.0%	0	0.0%	1	2.7%
	Other	0	0.0%	1	5.0%	0	0.0%	1	2.7%
**Who do you trust to buy you insurance? Other**	Banks	2	1.6%	4	2.6%	1	2.1%	7	2.2%
	Pakistan post	0	0.0%	2	1.3%	0	0.0%	2	0.6%
	Private Insurance company	6	4.8%	12	7.9%	3	6.4%	21	6.5%
	Government Insurance Company	0	0.0%	2	1.3%	4	8.5%	6	1.9%

These outcomes are complemented by the previous research showing a positive association between education and farmers’ willingness to get crop insurance [36,37]. Similarly, at the country level, a parallel stream of literature highlights the importance of education in mitigating environmental degradation and promoting economic growth [38,39].

Approximately 83% of respondents expressed that the government should provide crop insurance to protect against weather-related events and natural disasters. This finding aligns with existing literature that underscores the crucial role of government in subsidizing agricultural insurance in developing countries [21]. Similarly, Menash et al. [40] report that farmers in Ghana remain reluctant to adopt crop insurance without such support. In contrast, countries like China—where agricultural insurance premiums are heavily subsidized—have witnessed remarkable growth in insurance markets [41].

In contexts where farmers struggle for economic survival, allocating scarce resources to crop insurance is often perceived as a luxury rather than a necessity. Consequently, state subsidies emerge as the most effective means of making agricultural insurance products both accessible and attractive to farming communities.

The respondents were later probed to describe which types of crops they wanted insured. The most frequently mentioned crop category was cereal or grains, reported by 77%, followed by fiber crops. These crops constitute the major component of their annual output and, therefore, any damage to them causes a major setback for the rural economy.

Regarding willingness to pay, respondents indicated a maximum insurance contribution of Pakistan rupees 1,403 per season per acre of land. Respondents in Gujrat showed a willingness to contribute a higher premium compared to those in Bahawalpur and Faisalabad, as shown in Table 5.

While analyzing the same data by level of education, the only visible trend was respondents’ willingness to pay a per-acre insurance amount. Illiterate respondents considered paying PKR 973 per acre as insurance premium, while respondents with an education level up to matriculation considered a relatively higher amount of PKR 1,452. However, respondents with an intermediate and higher level of education were willing to pay 2,330 Pakistani rupees per acre as insurance premium, which is more than double what illiterate respondents were considering.

This indicates that respondents who are more aware of potential threats and their impact are willing to pay relatively more in insurance premiums compared to those who cannot properly understand and quantify the impacts. This result is further evidence of a positive association between human capital and individuals’ willingness to pay for crop insurance [42–44].

### 5.4. Respondent borrowing practices

Another important aspect of our investigation was to assess farmers’ reliance on financial assistance from both formal and informal sources to sustain their agricultural activities. As shown in Table 11, nearly two-thirds of respondents reported borrowing money or acquiring agricultural inputs on credit for crop cultivation. Borrowing practices, however, vary considerably across districts: farmers in Bahawalpur and Gujrat reported higher levels of borrowing compared to those in Faisalabad. The form of borrowing also differs, with cash loans being more common in Gujrat, while in-kind borrowing was more frequently observed in Bahawalpur. Among those who borrow, almost half rely on input suppliers, whereas about 22% depend on commission agents or middlemen, highlighting the central role of informal credit channels in financing agricultural production.

**Table 11:** Respondents’ borrowing practices by district.

		Bahawalpur		Faisalabad		Gujrat		Overall	
		N	%age	N	%age	N	%age	N	%age
**Do you borrow money/inputs for crop cultivation?**	Yes (in cash)	6	5.6%	19	17.6%	33	30.6%	58	17.9%
	Yes (in kind)	60	55.6%	34	31.5%	21	19.4%	115	35.5%
	Both (in cash and in-kind)	11	10.2%	14	13.0%	21	19.4%	46	14.2%
	No	31	28.7%	41	38.0%	33	30.6%	105	32.4%
**What is the source of borrowing?**	Commission agent/middleman	9	11.7%	10	14.9%	28	37.3%	47	21.5%
	Beopari	4	5.2%	4	6.0%	4	5.3%	12	5.5%
	Input shops	58	75.3%	37	55.2%	16	21.3%	111	50.7%
	Landlord	1	1.3%	10	14.9%	12	16.0%	23	10.5%
	Bank	12	15.6%	7	10.4%	8	10.7%	27	12.3%
	Other	3	3.9%	11	16.4%	16	21.3%	30	13.7%
**Why do you prefer informal sources over borrowing from a bank?**	Trust relationship	8	11.3%	3	4.8%	17	23.9%	28	13.7%
	Easiness (no documentation)	33	46.5%	7	11.1%	15	21.1%	55	26.8%
	Easy approach	48	67.6%	43	68.3%	39	54.9%	130	63.4%
	Direct payment	23	32.4%	4	6.3%	3	4.2%	30	14.6%
	Fixed interest/markup	6	8.5%	1	1.6%	1	1.4%	8	3.9%
	Low interest rate	8	11.3%	8	12.7%	7	9.9%	23	11.2%
	No interest rate	4	5.6%	14	22.2%	9	12.7%	27	13.2%
	Other	2	2.8%	0	0.0%	4	5.6%	6	2.9%
**For what purpose is the borrowing used?**	Purchase of seed	46	59.7%	59	88.1%	53	70.7%	158	72.1%
	Purchase of fertilizers	72	93.5%	65	97.0%	68	90.7%	205	93.6%
	Purchase of pesticides	71	92.2%	49	73.1%	38	50.7%	158	72.1%
	Renting a tractor for cultivation	2	2.6%	1	1.5%	4	5.3%	7	3.2%
	Renting harvesting machinery	0	0.0%	5	7.5%	9	12.0%	14	6.4%
	Hiring Labor	1	1.3%	3	4.5%	4	5.3%	8	3.7%
	To pay electricity/ diesel bills	5	6.5%	13	19.4%	20	26.7%	38	17.4%
	Non-agricultural use	2	2.6%	4	6.0%	6	8.0%	12	5.5%
	Other	0	0.0%	3	4.5%	3	4.0%	6	2.7%

Given that most respondents borrowed from input shops or commission agents/middlemen, they were further asked why they preferred informal credit sources over banks. The most frequently cited reason was the “easy approach,” reported by 64% of respondents, followed by the absence of documentation requirements, mentioned by 27%. Notably, the ease of borrowing without formal paperwork was reported more often in Bahawalpur compared to other districts. The borrowed funds were primarily used for the purchase of essential agricultural inputs such as seeds, fertilizers, and pesticides.

Table 12 summarizes the respondents’ borrowing practices by education level. Generally, respondents with a higher level of education borrow less both in cash and in-kind, and they prefer to borrow from commission agents or middlemen, rather than from input shops. Reasons for choosing informal borrowing sources and the purpose of borrowing are similar across respondents’ level of education.

**Table 12:** Respondents’ borrowing practices by respondent level of education.

		Illiterate		Up to Matriculation		Intermediate & above		Total	
		N	%age	N	%age	N	%age	N	%age
**Do you borrow money/inputs for crop cultivation?**	Yes (in cash)	22	17.5%	27	17.9%	9	19.1%	58	17.9%
	Yes (in kind)	50	39.7%	52	34.4%	13	27.7%	115	35.5%
	Both (in cash and in kind)	21	16.7%	18	11.9%	7	14.9%	46	14.2%
	No	33	26.2%	54	35.8%	18	38.3%	105	32.4%
**What is the source of borrowing?**	Commission agent/ middleman	14	15.1%	22	22.7%	11	37.9%	47	21.5%
	Beopari	4	4.3%	5	5.2%	2	6.9%	11	5.0%
	Contractors	0	0.0%	1	1.0%	0	0.0%	1	0.5%
	Input shops	55	59.1%	45	46.4%	11	37.9%	111	50.7%
	Landlord	11	11.8%	9	9.3%	3	10.3%	23	10.5%
	Bank	13	14.0%	10	10.3%	4	13.8%	27	12.3%
	Moneylenders (Bunia, Seth, Memon)	0	0.0%	0	0.0%	0	0.0%	0	0.0%
	Other	12	12.9%	14	14.4%	4	13.8%	30	13.7%
	9	0	0.0%	2	2.1%	0	0.0%	2	0.9%
**Why do you prefer informal sources over borrowing from a bank?**	Trust relationship	13	14.8%	11	12.1%	4	15.4%	28	13.7%
	Ease (no documentation)	26	29.5%	24	26.4%	5	19.2%	55	26.8%
	Easy approach	58	65.9%	57	62.6%	15	57.7%	130	63.4%
	Direct payment	17	19.3%	11	12.1%	2	7.7%	30	14.6%
	Fixed interest/ markup	3	3.4%	3	3.3%	2	7.7%	8	3.9%
	Low interest rate	9	10.2%	8	8.8%	6	23.1%	23	11.2%
	No interest rate	10	11.4%	13	14.3%	4	15.4%	27	13.2%
	Other	2	2.3%	3	3.3%	1	3.8%	6	2.9%
**For what purpose is the borrowing used?**	Purchase of seed	64	68.8%	72	74.2%	22	75.9%	158	72.1%
	Purchase of fertilizers	87	93.5%	91	93.8%	27	93.1%	205	93.6%
	Purchase of pesticides	67	72.0%	70	72.2%	21	72.4%	158	72.1%
	Renting a tractor for cultivation	3	3.2%	4	4.1%	0	0.0%	7	3.2%
	Renting harvesting machinery	2	2.2%	8	8.2%	3	10.3%	13	5.9%
	Renting threshing machinery	0	0.0%	1	1.0%	0	0.0%	1	0.5%
	Hiring Labor	3	3.2%	4	4.1%	1	3.4%	8	3.7%
	To pay electricity/ diesel bills	11	11.8%	21	21.6%	6	20.7%	38	17.4%
	Non-agricultural use	6	6.5%	6	6.2%	0	0.0%	12	5.5%
	Other	2	2.2%	3	3.1%	1	3.4%	6	2.7%

It is generally assumed that farmers excluded from formal credit markets are those who lack sufficient collateral to secure loans. Our subsequent investigation sought to test this theoretical prediction. As shown in Table 13, farmers reported preferring informal sources of credit primarily because they either require no collateral or accept only personal guarantees. Nearly three-quarters of respondents indicated that no guarantee was needed to borrow from the informal sector, while others relied on personal guarantees provided by a well-off relative or friend. Repayment was typically made either in cash or in kind, reflecting the flexible nature of informal credit arrangements.

**Table 13:** Guarantee to avail of informal borrowing and return plans by district.

		Bahawalpur		Faisalabad		Gujrat		Overall	
		N	%age	N	%age	N	%age	N	%age
**Do you need any kind of guarantee to avail of borrowing from informal loan providers?**	Yes	23	31.9%	13	20.6%	16	22.5%	52	25.2%
	No	49	68.1%	50	79.4%	55	77.5%	154	74.8%
**What kind of guarantee is required by the informal loan providers?**	Yes – Personal guarantee by a well-off relative	10	35.7%	7	41.2%	6	30.0%	23	35.4%
	Yes – Personal guarantee by a friend	11	39.3%	8	47.1%	5	25.0%	24	36.9%
	Yes – Guarantee that you sell back your produce to the lender	2	7.1%	1	5.9%	4	20.0%	7	10.8%
	Other	10	35.7%	2	11.8%	5	25.0%	17	26.2%
	No guarantee is needed	0	0.0%	0	0.0%	0	0.0%	0	0.0%
**What is the mode of repayment of loans borrowed from the informal sector (commission agent, Beopari, landlord, input shops, contractor)**	Through cash	57	52.8%	40	37.0%	21	19.4%	118	36.4%
	In-kind (commodity against loan)	18	16.7%	15	13.9%	32	29.6%	65	20.1%
	Other	0	0.0%	0	0.0%	0	0.0%	0	0.0%

### 5.5. Respondents’ experience with the banking sector

To complete the picture, the research probed respondents about their overall experience with the banking sector. The purpose was to understand respondents’ access, exposure, and perception of various banking services so we could understand whether this is a potential contributor or barrier to farmers’ access to crop insurance schemes.

The results show that a little more than one-third of respondents (36%) had no experience with the banking sector, while almost 30% had deposit accounts, about 23% went to the bank for utility payments and about 20% used automated teller machine (ATM) cards. Nearly 85% of respondents rated their overall experience with the banking sector as ‘very good’ or ‘good’.

More respondents in Gujrat used banking services compared to Bahawalpur and Faisalabad. These outcomes reveal geographical disparities between south and central Punjab which may be associated with difference in income and education levels. It is encouraging to see that mobile banking (easy paisa, jazz cash, etc.) was consistent across sampled districts from Northern and Southern Punjab. This provides a possible engagement opportunity for financial transactions and services.

Table 14 examines the correlation between respondents’ level of education and their experience with banking services. As anticipated, the use of banking services is strongly correlated with respondents’ education levels. More than half of illiterate respondents reported never having accessed banking services, compared to 33% of those with education up to the matriculation level. In contrast, only 6.4% of respondents with an intermediate or higher level of education reported having no experience with banking services.

**Table 14:** Respondents’ exposure and experience of banking services by district.

		Illiterate		Up to Matriculation		Intermediate & above		OVERALL	
		N	%age	N	%age	N	%age	N	%age
**What banking services have you used?**	No experience	65	51.6%	49	32.5%	3	6.4%	117	36.1%
	Deposit account	17	13.5%	52	34.4%	28	59.6%	97	29.9%
	Loan account	23	18.3%	18	11.9%	3	6.4%	44	13.6%
	ATM	16	12.7%	34	22.5%	13	27.7%	63	19.4%
	Online banking	0	0.0%	4	2.6%	2	4.3%	6	1.9%
	Mobile banking (easy paisa, jazz cash, etc.)	8	6.3%	19	12.6%	15	31.9%	42	13.0%
	Utilities payment	24	19.0%	33	21.9%	17	36.2%	74	22.8%
	Funds transfer	0	0.0%	16	10.6%	4	8.5%	20	6.2%
	Other	1	0.8%	4	2.6%	3	6.4%	8	2.5%
**What is your opinion about the overall banking experience?**	Very good	3	4.9%	4	3.9%	3	6.8%	10	4.8%
	Good	45	73.8%	86	84.3%	34	77.3%	165	79.7%
	Poor	13	21.3%	12	11.8%	7	15.9%	32	15.5%

Furthermore, illiterate respondents were more likely to report unfavorable experiences with banks compared to their literate counterparts. These results are consistent with the broader finance literature highlighting the role of education in fostering financial inclusion and enabling farming communities to engage more effectively with formal financial institutions [45,46].

Another dimension of inquiry examined respondents’ experiences with bank loans. When asked whether they had applied for a loan in the past three years, about 24% reported doing so, with the share of applicants in Bahawalpur nearly double that of the other districts. Among those who applied, nearly 95% successfully secured loans, indicating that the main barrier to financial inclusion is not credit availability or loan approval but a limited awareness and engagement with formal financial institutions.

The remaining 76% of respondents who had not applied for a loan in the past three years were further asked about their reasons. Nearly half (45%) stated that they did not feel the need to borrow, while 20% considered bank loans too expensive. Religious concerns also played a role, with 19% citing the prohibition of interest (markup) in Islam as a reason for non-participation. Another 16% pointed to the lengthy and complex loan application process.

Among those who applied for a loan, just over half sought regular bank financing, while 18% applied under the *Kissan Dost* schemes. Overall, around 60% of loan recipients expressed satisfaction with the bank loan process.

A higher proportion of educated respondents reported applying for bank loans in the past three years compared to illiterate respondents. Interestingly, however, loan approval rates were higher among illiterate respondents and those with up to matriculation qualifications. Respondents with intermediate education or above were more likely to perceive bank loans as expensive than their less educated counterparts.

This may be attributed to their greater capacity to compare bank loan markups with alternative borrowing options, allowing them to critically assess the relative cost of formal credit. It is worth mentioning that interest rates in Pakistan remained high over the past few years. This has presented a major obstacle to farmers seeking credit through formal banking channels.

Multiple data points highlight the same constraints beyond individual traits: low baseline awareness (20.7%; Table 4); dominance of informal credit channels over banks (Table 11); administrative frictions in bank processes cited by non-applicants (Table 15); premium affordability and product-fit concerns alongside limited coverage of non-major crops (Tables 5 and 6 and program descriptions); religious constraints (18.8%; Tables 15 and 16); and limited banking experience for over one-third of respondents (Table 17).

**Table 15:** Respondents’ experience and satisfaction with bank loans by district.

		Bahawalpur		Faisalabad		Gujrat		Overall	
		N	%age	N	%age	N	%age	N	%age
**Have you applied for a Bank loan in the last three years?**	Yes	31	54.4%	21	28.0%	27	36.0%	79	24.4%
**What is the status of the loan applied to the Bank?**	Approved	31	100.0%	20	95.2%	24	88.9%	75	94.9%
	Not approved	0	0.0%	1	4.8%	2	7.4%	3	3.8%
	Still pending	0	0.0%	0	0.0%	1	3.7%	1	1.3%
**Why don’t you apply to the Bank for a loan?**	No need	14	56.0%	26	48.1%	18	36.7%	58	45.3%
	Lack of information/ processes	2	8.0%	3	5.6%	4	8.2%	9	7.0%
	Lengthy process for loan application	6	24.0%	6	11.1%	9	18.4%	21	16.4%
	Don’t have complete documents	0	0.0%	1	1.9%	0	0.0%	1	0.8%
	Loans are too expensive	3	12.0%	13	24.1%	10	20.4%	26	20.3%
	Fear of loose collateral	2	8.0%	3	5.6%	3	6.1%	8	6.3%
	Markup/interest is forbidden in religion	3	12.0%	8	14.8%	13	26.5%	24	18.8%
**What type of financing/ loans have you applied for in the last three years?**	Kissan Dost Scheme	3	9.7%	6	28.6%	5	18.5%	14	17.7%
	Sada Bahar Scheme	1	3.2%	1	4.8%	2	7.4%	4	5.1%
	Provincial govt. Markup-free Agri-credit Scheme	0	0.0%	0	0.0%	2	7.4%	2	2.5%
	Financing scheme for “Dairy value chain”	0	0.0%	1	4.8%	2	7.4%	3	3.8%
	Any Other scheme (specify)	1	3.2%	4	19.0%	5	18.5%	10	12.7%
	Running Finance	3	9.7%	0	0.0%	2	7.4%	5	6.3%
	Regular Bank financing	24	77.4%	9	42.9%	9	33.3%	42	53.2%
**Were you satisfied with the bank loan process?**	Yes	22	71.0%	12	57.1%	13	48.1%	47	59.5%
	No	9	29.0%	9	42.9%	14	51.9%	32	40.5%

**Table 16:** Respondents’ experience and satisfaction with bank loans by level of education.

		Illiterate		Up to Matriculation		Intermediate & above		Overall	
		N	%age	N	%age	N	%age	N	%age
**Have you applied for a Bank loan in the last three years?**	Yes	28	22.2%	38	25.2%	13	27.7%	79	24.4%
**What is the status of the loan applied to the Bank?**	Approved	27	96.4%	36	94.7%	12	92.3%	75	94.9%
	Not approved	1	3.6%	2	5.3%	0	0.0%	3	3.8%
	Still pending	0	0.0%	0	0.0%	1	7.7%	1	1.3%
**Why don’t you apply to the Bank for a loan?**	No need	16	48.5%	29	45.3%	13	41.9%	58	45.3%
	Lack of information/ processes	4	12.1%	5	7.8%	0	0.0%	9	7.0%
	Lengthy process for loan application	2	6.1%	15	23.4%	4	12.9%	21	16.4%
	Don’t have complete documents	0	0.0%	0	0.0%	1	3.2%	1	0.8%
	Loans are too expensive	6	18.2%	10	15.6%	10	32.3%	26	20.3%
	Fear of loose collateral	4	12.1%	2	3.1%	2	6.5%	8	6.3%
	Markup/interest is forbidden in religion	3	9.1%	15	23.4%	6	19.4%	24	18.8%
	Other	0	0.0%	0	0.0%	0	0.0%	0	0.0%
**What type of financing/ loans have you applied for in the last three years?**	Kissan Dost Scheme	4	14.3%	7	18.4%	3	23.1%	14	17.7%
	Sada Bahar Scheme	1	3.6%	3	7.9%	0	0.0%	4	5.1%
	Provincial govt. Markup-free Agri-credit Scheme	0	0.0%	1	2.6%	1	7.7%	2	2.5%
	Financing scheme for “Dairy value chain”	0	0.0%	2	5.3%	1	7.7%	3	3.8%
	Any Other scheme (specify)	3	10.7%	7	18.4%	0	0.0%	10	12.7%
	Running Finance	2	7.1%	1	2.6%	2	15.4%	5	6.3%
	Regular Bank financing	19	67.9%	17	44.7%	6	46.2%	42	53.2%
**Were you satisfied with the bank loan process?**	No	11	39.3%	18	47.4%	3	23.1%	32	40.5%
	Yes	17	60.7%	20	52.6%	10	76.9%	47	59.5%

**Table 17:** Respondents’ exposure and experience of banking services by district.

		Bahawalpur		Faisalabad		Gujrat		Overall	
		N	%age	N	%age	N	%age	N	%age
**What banking services have you used?**	No experience	51	47.2%	33	30.6%	33	30.6%	117	36.1%
	Deposit account	24	22.2%	33	30.6%	40	37.0%	97	29.9%
	Loan account	28	25.9%	11	10.2%	5	4.6%	44	13.6%
	ATM	19	17.6%	22	20.4%	22	20.4%	63	19.4%
	Online banking	1	0.9%	0	0.0%	5	4.6%	6	1.9%
	Mobile banking (easy paisa, etc.)	14	13.0%	13	12.0%	15	13.9%	42	13.0%
	Utility payment	1	0.9%	47	43.5%	26	24.1%	74	22.8%
	Funds transfer	2	1.9%	10	9.3%	8	7.4%	20	6.2%
	Other	0	0.0%	4	3.7%	4	3.7%	8	2.5%
**Opinion about overall banking experience?**	Very good	2	3.5%	2	2.7%	6	8.0%	10	4.8%
	Good	43	75.4%	61	81.3%	61	81.3%	165	79.7%
	Poor	12	21.1%	12	16.0%	8	10.7%	32	15.5%

Together, these suggest that the underperformance of crop insurance reflects systemic frictions in information, distribution, process, design, and institutional trust rather than farmer-specific deficits.

### 5.6. Understanding factors that influence the adoption of crop insurance

The study employed regression analysis for each outcome of interest to determine the strength of any association between outcomes (crop insurance awareness) and relevant independent variables (demographic profile, education, land holding, premium amount and experience with banking services and loans, etc.). Logistic regression (logit) is usually used when the dependent variable is dichotomous. Logistic regression measures the relationship between the dichotomous dependent variable and one or more independent variables by estimating probabilities using a logistic function. Logit is helpful for situations in which we want to predict the presence or absence of a characteristic or outcome based on the values of a set of independent (or predictor) variables.

As outlined in earlier sections, around 20% of respondents know about crop insurance, 10% of them had some form of insurance at the time of the survey, and only 3% had crop insurance. Since crop insurance adoption is very low, it does not become a useful indicator for drawing any meaningful association between independent and dependent variables. Therefore, the study uses crop insurance awareness (the highest possible outcome) as the dependent variable in the regression models.

The correlation matrix, reported in Table 7, does not reveal a significant association between crop insurance and our set of independent variables.

The results from the logit and probit regression models reveal several noteworthy insights into farmers’ perceptions and awareness of crop insurance. As reported in Table 18, and relative to Faisalabad (the reference category), farmers residing in Bahawalpur and Gujrat exhibit significantly higher awareness of crop insurance schemes. Moreover, the coefficient on education is positive and statistically significant, underscoring the critical role of human capital in promoting insurance awareness among farmers. More educated individuals are likely to have a better understanding of climate-related risks and therefore demonstrate a stronger preference for hedging against such systemic risks [47].

**Table 18:** Factors determining the perception of farmers about crop insurance.

Dep. Var.	(1)	(2)	(3)	(4)	(5)	(6)	(7)	(8)	(9)	(10)
(AWR)	Logit	Logit	Logit	Logit	Logit	Probit	Probit	Probit	Probit	Probit
D.BAH	1.170***	1.323***	1.281***	1.264***	1.457***	1.170***	1.323***	1.281***	1.264***	1.457***
	(0.373)	(0.382)	(0.385)	(0.387)	(0.398)	(0.373)	(0.382)	(0.385)	(0.387)	(0.398)
D.GUJ	0.827**	0.846**	0.803**	0.696*	0.726*	0.827**	0.846**	0.803**	0.696*	0.726*
	(0.384)	(0.386)	(0.388)	(0.395)	(0.401)	(0.384)	(0.386)	(0.388)	(0.395)	(0.401)
EDU		0.744**	0.701**	0.601*	0.424		0.744**	0.701**	0.601*	0.424
		(0.352)	(0.355)	(0.359)	(0.368)		(0.352)	(0.355)	(0.359)	(0.368)
LH			0.006	0.007	0.006			0.006	0.007	0.006
			(0.006)	(0.006)	(0.006)			(0.006)	(0.006)	(0.006)
PREM				0.001**	0.001**				0.001**	0.001**
				(0.000)	(0.000)				(0.000)	(0.000)
BANK					1.215***					1.215***
					(0.365)					(0.365)
Constant	−2.079***	−2.709***	−2.739***	−2.798***	−3.580***	−2.079***	−2.709***	−2.739***	−2.798***	−3.580***
	(0.306)	(0.437)	(0.439)	(0.441)	(0.524)	(0.306)	(0.437)	(0.439)	(0.441)	(0.524)
Observations	324	324	324	324	324	324	324	324	324	324

Standard errors in parentheses: *** p < 0.01, ** p < 0.05, * p < 0.1

In contrast, landholding size does not appear to have a statistically significant effect on insurance awareness. Interestingly, the insurance premium variable shows a positive association with insurance perception, suggesting that better-informed farmers are more likely to recognize the value of insurance coverage and are consequently willing to pay higher premiums. Finally, the use of banking services is positively and significantly associated with insurance awareness, indicating that farmers with greater integration into the formal financial system are more likely to be informed about available agricultural insurance schemes. Consistent with these findings, prior studies document a strong feedback relationship between financial inclusion and individuals’ acceptance of insurance products [48].

Overall, these findings underscore the importance of financial, educational, and geographical factors in shaping individuals’ awareness of crop insurance. While the regression analysis incorporated a broader set of explanatory variables, as discussed earlier, none of these additional factors were statistically significant in the selected logit and probit specifications. The adjusted R-squared values, ranging from 0.30 to 0.50, suggest that the included variables explain a substantial share of the variation in crop insurance knowledge.

## 6. Conclusions and policy recommendations

This study highlights a significant gap in awareness and uptake of crop insurance among rural households in Punjab. Despite the availability of both government and private initiatives, 79% of surveyed farmers reported no knowledge of such programs, and fewer than 3% had ever participated. Even though the majority recognized climate-related risks—particularly pests, diseases, floods, droughts, and extreme heat—most continued to rely on informal credit networks due to their accessibility, flexibility, and trust-based nature. Borrowing patterns revealed deep financial vulnerability: nearly two-thirds depended on informal loans for agricultural inputs, while over one-third had no banking experience. Regression models examining demographic and financial variables found that education, geography and financial inclusion had significant effects as predictors of insurance awareness. Profiling of the small subgroup familiar with crop insurance showed no consistent trends across education, landholding, or location.

Overall, the findings reveal limited understanding of the purpose, benefits, and processes of crop insurance, reflecting weaknesses in dissemination and institutional credibility. Addressing these gaps requires farmer-centric strategies, including community-based awareness, streamlined enrolment, and stronger institutional trust, to transform crop insurance into an effective and inclusive tool for climate resilience in Pakistan’s agricultural sector.

To translate the study’s findings into actionable changes, the following policy and programmatic recommendations are offered. First, insurance awareness should be systematically integrated into agricultural extension services, where trained officers, cooperatives, and community leaders act as trusted intermediaries to build financial literacy and grassroots confidence. Complementing this, targeted, region-specific awareness campaigns delivered through local communication channels such as radio and farmer groups can demystify insurance processes using culturally relevant messages. At the institutional level, fostering public–private partnerships (PPPs) can combine government-backed credibility and subsidies with private sector innovation, ensuring wider outreach and operational efficiency. In parallel, digitizing and simplifying enrolment and claims procedures—through mobile platforms, satellite-based verification, and bundled financial products—can reduce administrative barriers. Finally, investment in robust data systems, including weather stations, yield monitoring, and cadastral mapping, alongside stronger inter-agency coordination, will be crucial for supporting parametric and area-yield insurance schemes. Future research should examine the socio-cultural and gender-specific determinants influencing the adoption of crop insurance among vulnerable communities, with particular attention to social norms, intra-household decision-making, and access to information in shaping farmers participation.

## Supporting information

S1 File Data set. (XLSX)
